# Comparative Efficacy of Cow Milk, KNO3, and Warm Saline Rinses in Treating Dentin Hypersensitivity Following Nonsurgical Periodontal Treatment: A Randomized Controlled Trial

**DOI:** 10.7759/cureus.12466

**Published:** 2021-01-04

**Authors:** Charu Mohan Marya, Sonam Jain, Ruchi Nagpal, Sakshi Kataria, Pratibha Taneja, Sunny Mavi

**Affiliations:** 1 Public Health Dentistry, Sudha Rustagi College of Dental Sciences & Research, Faridabad, IND; 2 Periodontology, Sudha Rustagi College of Dental Sciences & Research, Faridabad, IND

**Keywords:** milk proteins, caseins, dentin hypersensitivity, scaling and root planing

## Abstract

Aim: This study aimed to assess the desensitizing efficacy of commercially available cow milk compared to potassium nitrate (KNO_3_) mouthwash and warm saline rinses after nonsurgical periodontal treatment.

Materials and Methods: A randomized controlled trial was conducted in the Department of Public Health Dentistry of a dental college from August 21, 2018 to September 10, 2018. A total of 75 patients who reported hypersensitivity after scaling and root planing (SRP) were recruited and randomly assigned into three groups: cow milk (I), KNO_3_ mouthwash (II), and warm saline rinses (III). Dentine hypersensitivity (DH) was assessed at six time points using the verbal rating scale (VRS) for thermal stimuli and visual analog scale (VAS) for air blast and thermal stimuli. Statistical analysis was done using Statistical Package for Social Sciences (SPSS) version 21 (IBM Corp., Armonk, NY). Mean reduction in DH in each group was measured using non-parametric tests.

Results: Patients in all the three groups were found to be comparable with respect to baseline characteristics. Mean reduction in VRS and VAS scores for DH in milk and KNO_3_ mouthwash was found to be significantly high as compared to warm saline rinses group.

Conclusion: From the results, cow milk was found to be equivalent in efficacy as compared with KNO_3_ mouthwash but superior to warm saline rinses in treating DH post-SRP.

## Introduction

Scaling and root planing (SRP) is the mainstay of nonsurgical periodontal therapy that aids in the effective removal of bacterial deposits from the tooth surface. It prevents the initiation or progression of gingival and periodontal diseases [1]. It is often accompanied by several undesirable side effects such as gingival recession and exposure of root dentin due to the removal of cementum. This iatrogenic denudation of root dentin due to removal of the cementum layer may result in a large number of dentinal tubules to be exposed, which may serve as gateways from where bacteria may enter and approach pulp [2]. Consequently, it leads to increased sensitivity to external stimuli. This condition, when gets severe, has been termed as dentin hypersensitivity (DH), dentin sensitivity or root dentin sensitivity, or cervical dentin sensitivity in literature [3].

 DH is defined as pain derived from exposed dentin in response to chemical, thermal tactile, or osmotic stimuli, which cannot be explained as arising from any other dental defect or disease [4]. It is a relatively common problem faced by every dental clinician in daily practice. Its mechanism can be best explained through Brännström’s hydrodynamic theory. This theory proposes that there is a change in dentinal fluid flow in dentinal tubules, which is caused by pain-producing stimuli, thereby activating intra-dental nerve fibers via mechanoreceptors, which eventually causes pain [5].

SRP procedure is amenable for causing specific changes for DH to occur like exposure and denudation of dentin and enamel surface combined with the loss of cementum leading to the opening of the dentin tubules that stimulate sensory mechanisms in the pulpal area [6]. An evidence for the association of oral prophylaxis and DH has been documented in a study where 32% of patients experienced increased hypersensitivity after oral prophylaxis [7]. The advent of DH after this preventive treatment often makes people apprehensive of getting it done. Therefore, there is a need to address this situation to further prevent more severe sequelae of periodontal diseases. Treatment modalities for DH have been formulated, which either decrease the neural transmission or physically occlude the patent tubule [8]. A wide range of desensitizing agents are available as over-the-counter products such as toothpaste, mouth wash, or in-office therapy such as varnishes, dentin-bonding agents, and others [9]. But the most preferred material is KNO_3_, which is known to act on the nerves sensing pain in tooth [10].

Considering that the majority of the population in India resides in the rural areas (68.84%) [11], where oral care facilities are far below the required standards [12], these products or treatment modalities are not easily available. Hence, it is perceived that there is a need for a more acceptable treatment modality for DH. Acceptance of any agent could be amplified if it is familiar or easily available to a larger population. Recently milk protein “casein” has evolved as a remineralizing agent. A number of products are available in the market containing casein protein, namely, casein phosphopeptides-amorphous calcium phosphate (CPP-ACP) under various trade names. Because 80% of cow milk is composed of casein, studies [13,14] have been conducted to evaluate the remineralizing or desensitizing property of cow milk in reducing DH after SRP. These studies concluded that cow milk has considerable efficacy in reducing DH after SRP. Milk is an essential commodity in every Indian household, and if confirmed it can be a very promising public health intervention because of three As: affordability, ease of availability, and huge scope of acceptability. Its benefits can be attributed to the low content lactose for non-cariogenic and protective properties, limiting cariogenic potential and the high casein, calcium, and phosphate content that resists demineralization and aids remineralization of enamel and dentin [15].

To the best of authors’ knowledge of the present literature, there is a dearth of evidence assessing the desensitizing potential of cow milk after SRP. Hence, the present study was carried out with the null hypothesis stating that the efficacy of commercially available cow milk rinses is equivalent to potassium nitrate mouthwash and warm saline rinses in treating DH after nonsurgical periodontal therapy.

## Materials and methods

This was a single-center, randomized controlled trial of concurrent parallel design with three arms aimed to assess the desensitizing potential of cow milk as compared to KNO_3_ and warm saline rinses after SRP. It was conducted in the Department of Public Health Dentistry of Sudha Rustagi College of Dental Sciences and Research, Faridabad (Haryana, India), from August 21, 2018 to September 10, 2018. Outcome assessors and data analysts were kept blinded to the group allocation states of all patients. Ethical standards of the World Medical Association for human experimentation, 2013 version of the Helsinki Declaration, were followed throughout the study and were reported in accordance with the CONSORT guidelines [16]. This trial has been approved by the Institutional Ethical Committee (IEC) and has been prospectively registered in the Clinical Trials Registry India (CTRI) with registration number - CTRI/2018/08/015395 (August 20, 2018).

Clinical cases

A thorough clinical examination was done for the patients who reported complaint of DH after undergoing SRP in the Department of Public Health Dentistry. Eligibility criteria for the clinical cases/patients were age 20 years or above who were ready to give informed consent, systemically healthy, could understand and comply with the study protocol, and were residing within a 3-km radius of the institute. Patients who were undergoing or had a history of desensitizing therapy or history of periodontal surgery in the preceding three months or unrestored carious lesions, cervical abrasions, erosions, extensively restored teeth, impacted teeth with pain and ortho-appliances, crowns, bridges, restorations extending in the area of DH, or allergic to milk or test products were excluded. Also, the patients who were taking medications like analgesics and immunosuppressant as well as pregnant or lactating mothers were also excluded from the study. A written consent was obtained from the subjects who were willing to participate in the study.

Outcome measures

There were two primary outcome measures: verbal rating scale (VRS) scores for thermal stimuli and visual analogue scale (VAS) scores for air blast and thermal stimuli.

Thermal stimuli were applied by asking patient to rinse with water at room temperature and at 7˚C. Water at room temperature was provided first followed by water at 7˚C at an interval of 10 min. Air blast was applied through a three-way syringe (60-75 psi) to all teeth for duration of 1 s from approximately 1 cm away from the teeth. VRS is a four‑point scale to find out the numerical values of the clinical problem of DH. Its scores are as follows: Score 1 - no hypersensitivity, no discomfort to thermal changes after drinking water at room temperature or cold water; Score 2 - mild hypersensitivity, mild discomfort after drinking water at room temperature and cold water; Score 3 - moderate hypersensitivity, moderate discomfort after drinking water at room temperature but cannot drink cold water; and Score 4 - severe hypersensitivity, pain after drinking water at room temperature, pain on breathing, cannot tolerate cold water (severe pain). Response on VAS was recorded by asking the subject to mark on a 10-cm line labeled with no pain on one end and intolerable pain on the other along with facial expressions depicting the severity of pain for both the stimuli.

Sample size

Sample size estimation was done by using G*Power software (version 3.0, developed at Universität Düsseldorf in Germany). Sample size was estimated for mean. A minimum total sample size of 75 (25 in each group) was found to be sufficient for an alpha of 0.05, power of 80%, and 0.83 as effect size (assessed for the difference in VAS scores 15 days after starting of mouth rinses).

Randomization, group allocation, and intervention

The study investigator assessed the baseline score for DH (one day after the SRP procedure) for the included patients and randomly assigned them into three interventional groups in 1:1:1 ratio to receive commercially available cow milk, KNO_3_ mouth rinse, and advised warm saline rinses, respectively. Simple randomization was done using a random number list, which was created by a random number generator (QuickCalcs Online Random Numbers, GraphPad Software Inc., San Diego, CA). Intervention use was started a day after SRP. In group I, patients received commercially available cow milk that was boiled and cooled to room temperature, which was then filled in 30-mL bottles and dispensed daily to the patients (two each) for 21 days. It was done by the study investigator with the help of the departmental attendant who lived nearby. Patients were advised to rinse with 30-mL cow milk for two min twice daily for 21 days. In group II, patients were given mouthwash (Senquel-AD) with active agents as follows: potassium nitrate topical (3%) and sodium fluoride topical (0.2%). They were advised to rinse with 10 mL of mouthwash for one min twice daily (as per manufacturer’s recommendation) for 21 days. The bottle of mouthwash was given every week for 21 days containing 200 mL of mouthwash. In group III, patients were advised to rinse with 3 mL of warm saline water for two min twice daily for 21 days. All the patients were given uniform instructions to prepare it to make it standardized. They were told to add one teaspoon of commonly available table salt in 200 mL of lukewarm water and stir until the salt dissolved. During the study, patients were advised not to eat or drink for 30 min after the rinse and not to use any other dental products.

Follow-up

Patients in all the three groups were instructed to return for follow-up at seventh, 14th, and 21st day of treatment. DH score on VRS for thermal stimuli and VAS score for both the stimuli were recorded on all the follow-up visits by co-investigator who was unaware of the allocation status of the patients.

Compliance

To ensure the compliance, patients in all the groups were given daily reminders through phone calls or WhatsApp messenger. Patients in group II were told to return back the bottle along with the remaining quantity at seventh, 14th, and 21st day of treatment.

Statistical analysis

Data were analyzed using Statistical Package for Social Sciences (SPSS) version 21 (IBM Corp., Armonk, NY). Continuous variables like age, VRS score for thermal stimuli, and VAS scores for thermal stimuli and air blast were summarized as mean and standard deviation (SD). Owing to the ordinal nature of the study outcome variable, i.e., VRS scores and VAS scores, non-parametric tests of significance (Kruskal-Wallis test and Friedman test) were used for intragroup and intergroup comparisons. The level of statistical significance was set at ≤0.05.

## Results

**Figure 1:**
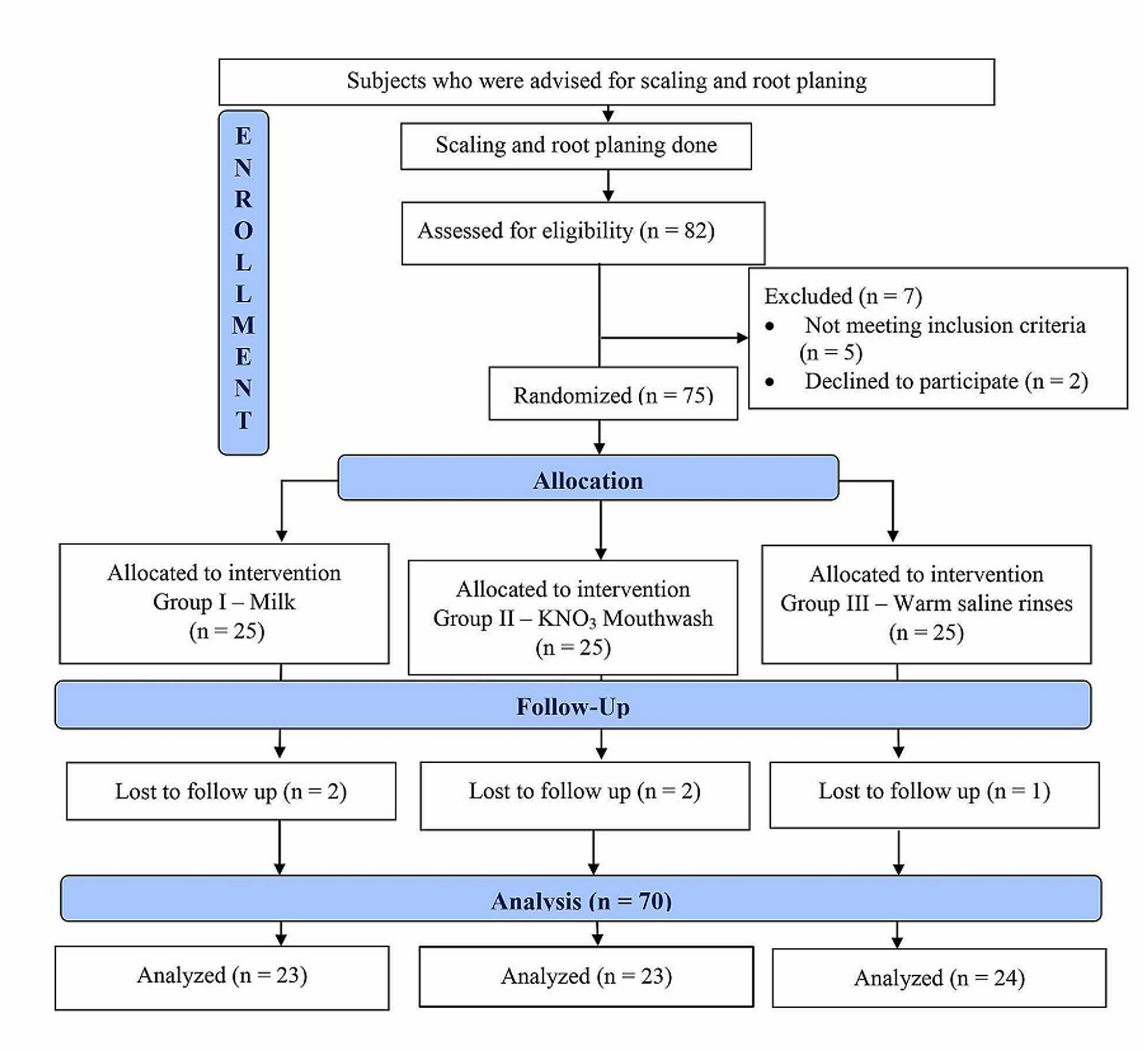
Flow chart of clinical cases recruitment

A total of 82 subjects were screened, seven did not fulfill eligibility criteria. Five participants dropped out for participants who completed all the follow-up visits (Figure 1). Mean age of the participants was 31.71 ± 7.9 years (42 males and 28 females). Mean periodontal probing depth (PPD) score at baseline was found to be significantly lower in group III as compared to group I and group II. Mean clinical attachment loss (CAL) score did not show any significant difference among all the groups (Table 1).

**Table 1:** Intragroup comparison of mean PPD and CAL scores at baseline ^a^Kruskal–Wallis test, ^b^Mann–Whitney U test, *Statistically significant (p value ≤ 0.05). PPD, Periodontal probing depth; CAL, clinical attachment loss.

	Group I	Group II	Group III	Total	p^a^-value	Post-hoc^b^
PPD (mean ± SD)	1.73 ± 0.25	1.89 ± 0.43	1.47 ± 0.21	1.69 ± 0.35	0.0001*	(I,II>III)
CAL (mean ± SD)	0.57 ± 1	0.49 ± 0.72	0.08 ± 0.08	0.37 ± 0.73	0.422	-

Mean absolute reduction of VRS and VAS scores for air blast and thermal stimuli were found to increase significantly from baseline to subsequent follow-up time points among all the three groups (Table 2).

**Table 2:** Intragroup comparison of mean absolute reduction of VRS and VAS scores of DH patients from baseline to follow-up visits ^a^Seventh day,^ b^14th day, ^c^21st day​​​​​​,​^ d^Friedman test, ^e^Wilcoxon signed-rank test, *Statistically significant (p value ≤ 0.05). VRS, Verbal rating scale; VAS, visual analog scale; DH, dentine hypersensitivity.

From baseline to follow-up visits		7^th^ day (a)	14^th^ day (b)	21^st^ day (c)	p^d^-value	Post-hoc^e^
Intervention group		Mean ± SD	Mean ± SD	Mean ± SD		
VRS scores (thermal stimuli)	Group I	0.17 ± 0.39	0.74 ± 0.54	1.09 ± 0.29	0.0001*	a < b < c
	Group II	0.35 ± 0.49	0.83 ± 0.39	1.17 ± 0.39	0.0001*	a < b < c
	Group III	0.33 ± 0.48	0.63 ± 0.49	0.96 ± 0.46	0.0001*	a < b < c
VAS scores (air blast)	Group I	0.91 ± 0.73	2.13 ± 1.18	3.26 ± 1.25	0.0001*	a < b < c
	Group II	1.04 ± 1.07	2.52 ± 1.5	3.65 ± 1.72	0.0001*	a < b < c
	Group III	0.83 ± 0.56	1.88 ± 0.95	3.17 ± 1.09	0.0001*	a < b < c
VAS scores (thermal stimuli)	Group I	1.48 ± 1.16	3.13 ± 1.42	4.57 ± 1.67	0.0001*	a < b < c
	Group II	1.74 ± 1.14	3.83 ± 1.27	5.04 ± 1.46	0.0001*	a < b < c
	Group III	1.04 ± 0.91	2.04 ± 1.08	3.25 ± 1.36	0.0001*	a < b < c

Intergroup comparison of mean percentage reduction of VRS and VAS scores for air blast and thermal stimuli showed that it was significantly more in group I and group II as compared to group III. No statistically significant difference in mean percentage reduction of VRS and VAS scores was found between group I and group II (Table 3).

**Table 3:** Intergroup comparison of mean percentage reduction of VRS and VAS scores of DH patients at follow-up visits ^a^Kruskal–Wallis test, ^b^Mann–Whitney U test, *statistically significant (p ≤ 0.05). VRS, Verbal rating scale; VAS, visual analog scale; DH, dentine hypersensitivity.

From baseline to follow-up visits	7th day			14th day			21st day		
	Group I	Group II	Group III	Group I	Group II	Group III	Group I	Group II	Group III
VRS scores (thermal stimuli)	6.52 ± 14.86	12.32 ± 17.56	10.7 ± 15.63	31.8 ± 22.98	34.78 ± 18.06	21.18 ± 17.20	48.55 ± 8.58	49.28 ± 10.63	34.72 ± 16.24
p^a^-value	0.040*			0.014*			0.0001*		
Post-hoc^b^	I,II>III			I,II>III			I,II>III		
VAS scores (air blast)	24.93 ± 23.01	24.44 ± 27.83	15.5 ± 13.18	58.33 ± 29.35	63.11 ± 34.52	34.94 ± 22.17	90.58 ± 17.10	86.98 ± 22.28	57.67 ± 24.81
p^a^-value	0.026*			0.001*			0.0001*		
Post-hoc^b^	I,II>III			I,II>III			I,II>III		
VAS scores (thermal stimuli)	30.23 ± 27.04	32.41 ± 20.74	17.45 ± 15.40	61.30 ± 28.82	73.10 ± 26.47	36.24 ± 20.25	87.02 ± 17.68	92.57 ± 14.66	58.02 ± 24.20
P^a^-value	0.043*			0.0001*			0.0001*		
Post-hoc pairwise analysis^b^	I,II>III			I,II>III			I,II>III		

## Discussion

The present study was a randomized controlled trial with three arms that assessed the efficacy of cow milk in treating DH following nonsurgical periodontal treatment. Although a double-blinded parallel-group design is best suited for conducting DH studies [17], due to practical constraints, patients could not be blinded. Therefore, the design of the study was drafted in a manner where the examiner was blinded who was assessing the DH scores on subsequent follow-up visits and was well-trained and calibrated under the guidance of experienced specialists. Allocation of the intervention was done by study investigator who was responsible for keeping records of the patients in all groups and keeping track of the follow-up visits. This was done to reduce allocation and observer bias in the study. Patients in all the three groups were homogenous in terms of mean age and gender. After a loss to follow-up of five patients, scores of 70 patients were analyzed.

In the studies in which desensitizing effect has been assessed, four-week exposure time has been widely used with a range of two to 12 weeks [7,10,13,14]. Contemplating this with feasibility, the present study was conducted for duration of three weeks. According to guidelines for the design and conduct of clinical trials [17] on DH, it is recommended that at least two hydrodynamic stimuli should be used and the least severe stimuli should be applied first. Controlled air blast and graded cold water stimuli (thermal stimuli) were used in the present study as they are physiological and controllable. Controlled air blasts, being less severe, was applied before the thermal stimuli keeping 10-min interval between them. This time interval was kept to minimize interaction between both the stimuli and quantifying maximally the effect of individual stimuli. Uniformity of the assessments for both the stimuli was maintained throughout the study. The use of prolonged evaporative stimuli has been criticized [18], and there is an evidence that if human dentin was dried with a stream of air for 5 min, it remained insensitive to painful stimuli, as long as it was kept dry [19]. On that account, air blast was applied with an air syringe for 1 s at a distance of 1 cm from the tooth surface to avoid desiccating of the dentin surface. Investigators have suggested that cold water at 7˚C was ideal for the identification of sensitive teeth as well as minimizing the incidence of false-positive responses [20]. Hence, water for thermal stimuli was maintained at 7˚C using TDS-TEMP meter (TDS3 TDS-3 Pocket TDS Meter, HM Digital, Inc., CA, USA).

DH was evaluated using the stimulus-based assessment and response-based assessment. VRS was used to assess the stimulus-based response, and their responses were recorded as mild, moderate, or severe DH. The response-based assessment was done using VAS.

In the present study, patients in first group were advised to rinse with commercially available cow milk at room temperature provided to them. Because there is no internationally recognized gold standard for treating DH, potassium nitrate has remained the most preferred agent [21]. Therefore, patients in the second group were advised to use Senquel-AD mouthwash containing KNO_3_as a positive control. In group III, warm saline rinses were advised to assess if removal of plaque due to rinsing action was the factor in decreasing DH and also it was used as a negative control as it is deprived of any active agent for the treatment of DH.

The VRS and VAS scores for DH decreased over the course of the follow-up period from the baseline (one day post-SRP), which were found to be comparable between milk and KNO_3_ group and higher in warm saline rinses group than the other two groups. This states that the milk rinses are equivalent to potassium nitrate in desensitization efficacy, and warm saline rinses had a minimum desensitizing effect. The result of this study is in accordance with the studies conducted by Madhurkar et al. [13] and Sabir et al. [14] where DH scores reduced on subsequent visits from baseline to 10th and 15th day, respectively.

Sabir et al. [14] proposed in their study that milk protein CPP contains phosphoryl sequences, which attach with amorphous calcium phosphate of teeth to form stabilized CPP-ACP further preventing dissolution of calcium and phosphate ions and maintain a supersaturated enamel lesions.

As it was a self-funded interest-based study, a smaller sample size was one of the limitations in generalizing the results of the present trial. Patient compliance and varied oral hygiene practices might have impact on the results of the study. The strength of the study lies in the fact that milk being universally accessible, affordable natural product did not require any safety trial.

## Conclusions

It can be concluded that cow milk is equivalent in efficacy as compared to KNO_3_ mouthwash but superior to warm saline rinses in treating DH post-SRP. Milk is a cheap and more acceptable mode of treatment after SRP. Still, there is a need to carry out further investigation to confirm the results and develop strategies for using milk products in order to prevent DH.
